# Spectroscopic quantification of cyclopentolate using an erythrosine-based resonance rayleigh scattering strategy: application to ophthalmic formulations

**DOI:** 10.1038/s41598-025-21762-4

**Published:** 2025-10-27

**Authors:** Ahmed A. Abu-hassan

**Affiliations:** Department of Pharmaceutical Analytical Chemistry, Faculty of Pharmacy, Al-Azhar University, Assiut Branch, Assiut, 71524 Egypt

**Keywords:** Cyclopentolate, RRS, Ion-pair, Ophthalmic formulations, Assay, Biological techniques, Diseases, Drug discovery, Medical research

## Abstract

Cyclopentolate (CYP), an antimuscarinic (anticholinergic) agent, is pharmacologically employed in ophthalmology to induce cycloplegia (temporary paralysis of the ciliary muscle) and mydriasis (pupil dilation) for diagnostic and therapeutic procedures, including ocular examinations and surgeries. This study establishes a spectrofluorimetric method for quantifying CYP in ophthalmic solutions based on Resonance Rayleigh Scattering (RRS) signal modulation. The approach exploits the formation of an ion-associate complex between CYP and the dye erythrosine, which induces a measurable RRS enhancement. Critical experimental parameters governing the complexation and subsequent RRS response were systematically investigated and optimized. Under these established optimal conditions, a linear correlation existed between the enhanced RRS intensity and CYP concentration across the range of 40 to 1500 ng/mL. The method demonstrated a detection limit (LOD) of 13 ng/mL and a quantification limit (LOQ) of 39.5 ng/mL. Successful application to the assay of cyclopentolate in commercial eye drop formulations confirmed the method’s accuracy and precision. This RRS-based methodology utilizing erythrosine presents a straightforward, rapid, cost-effective, and environmentally favorable option for routine quality control and monitoring of CYP in pharmaceutical preparations.

## Introduction

Cyclopentolate serves as a pivotal cycloplegic and mydriatic agent in ophthalmic practice, enabling critical diagnostic and therapeutic interventions for diverse ocular conditions. Its specific clinical utility encompasses refractive error assessment—particularly in pediatric hyperopia—alongside management of anterior uveitis, pseudomyopia, and pediatric myopia. Furthermore, cyclopentolate is indicated in the evaluation of idiopathic visual impairment and preoperative evaluations for refractive surgery^1^.

Cyclopentolate exerts its ophthalmic effects through competitive antagonism of muscarinic acetylcholine receptors. This mechanism inhibits parasympathetic input to the iris sphincter and ciliary musculature, resulting in unopposed sympathetic-mediated pupillary dilation (mydriasis) and paralysis of accommodation (cycloplegia). The induced mydriasis and loss of lens acclimatization facilitate enhanced visualization of intraocular structures during clinical examinations, thereby improving diagnostic precision^2^.

Notwithstanding its distinct therapeutic advantages over alternative cycloplegic agents, cyclopentolate administration entails clinically significant risks. Local adverse reactions—including ocular dysesthesia, photophobia, conjunctival hyperemia, punctate keratitis, synechiae formation, and transient visual disturbances—exhibit high incidence rates yet typically manifest with mild, self-limiting severity. Systemic sequelae, though uncommon, may encompass dysarthria, cerebellar ataxia, and visual or somatosensory hallucinations. These neuropsychiatric events predominantly occur in susceptible subpopulations. Furthermore, documented cases of non-therapeutic cyclopentolate utilization warrant consideration. While cyclopentolate remains a generally efficacious and safe pharmacological intervention, heightened vigilance is imperative given the potential for severe adverse events, particularly in vulnerable cohorts: pediatric and geriatric patients, individuals with neurological disorders or Down syndrome, pseudocholinesterase-deficient subjects, concurrent users of central nervous system depressants, and those with substance use disorders. Risk mitigation strategies include employing reduced cyclopentolate dosages—potentially co-administered with adjunctive mydriatics such as phenylephrine—and implementing nasolacrimal occlusion techniques post-instillation to reduce systemic absorption^3,4^.

Cyclopentolate is commercially formulated as topical ophthalmic solutions. Pharmacodynamic effects typically initiate within 15–60 min post-instillation, persisting for several hours with duration exhibiting concentration-dependent variability and significant interindividual variation. During the active cycloplegic phase, clinical manifestations commonly include accommodative paresis, photophobia, and impaired near vision secondary to ciliary muscle paralysis^5^.

Current analytical methodologies for cyclopentolate (CYP) quantification encompass spectrophotometric^6,7^, gas–liquid chromatographic^8^, potentiometric^9^, differential pulse polarographic^10^, and high-performance liquid chromatographic (HPLC)^11^ techniques. Critical evaluation reveals a paucity of established analytical protocols for CYP determination. Notably, despite the inherent advantages of spectrofluorimetry—including superior sensitivity, instrumental accessibility, and operational economy—no validated RRS method has been documented for CYP assay in pharmaceutical matrices. This methodological gap persists notwithstanding the technique’s demonstrated utility in quantifying structurally analogous ophthalmic agents.

Spectrofluorimetry represents a strategically significant analytical methodology for pharmaceutical quality control due to its multifaceted advantages. The technique achieves exceptional detection sensitivity, enabling reliable quantification of analytes at trace concentrations critical for pharmacopeial compliance. Furthermore, inherent molecular selectivity permits unambiguous target compound identification while mitigating matrix interference. Operational simplicity is evidenced by streamlined sample preparation protocols and minimal instrumental requirements, facilitating routine laboratory implementation. Cost efficiency relative to advanced analytical platforms provides economically viable solutions for resource-constrained environments. Given the ubiquitous deployment of spectrofluorimeters in analytical facilities, this technique offers readily deployable integration into established quality assurance frameworks, demonstrating compelling utility across diverse control applications^12–15^.

Erythrosine, an acidic xanthene dye, has been extensively employed in quantitative assays of basic pharmacotherapeutic agents including atomoxetine^16^, rasagiline^12^, duloxetine, and dapoxetine^17,18^. This analytical approach leverages electrostatic interactions between the dye’s anionic groups and protonated nitrogen moieties of basic drugs to form stable ion-pair complexes^19^. Subsequent quantification is achieved through multiple detection modalities: spectrophotometric analysis^20^, resonance Rayleigh scattering (RRS) techniques^21,22^, and spectrofluorimetry^23^. The erythrosine complexation method demonstrates exceptional sensitivity and selectivity for cationic drug molecules, producing chromogenic complexes amenable to spectroscopic monitoring. This versatile analytical strategy provides a robust framework for quality control validation of pharmaceutical formulations containing basic active constituents.

The increasing demand for environmentally sustainable, efficient, rapid, and highly responsive analytical methodologies for cyclopentolate (CYP) quantification necessitates innovative approaches. Spectrofluorimetry addresses these requirements through strategic utilization of fluorogenic dye interactions in acidic media. This investigation establishes a novel RRS-based assay employing erythrosine for ultrasensitive CYP determination. The methodology quantifies drug-induced RRS signal modulation arising from ion-associate complex formation between protonated CYP and erythrosine in buffered acidic solution. This approach demonstrates significant advantages over existing techniques: alignment with green analytical principles, operational efficiency (< 5 min analysis time), and user-friendly implementation without derivatization. Furthermore, the enhanced RRS responsiveness enables precise CYP quantification at nanogram levels with minimal matrix interference. Collectively, this erythrosine-RRS methodology provides a robust, sustainable solution for routine quality control of ophthalmic formulations.

## Experimental

### Apparatus

RRS intensity measurements were performed on a Sonico Luminescence Spectrometer equipped with a 150-W Xenon arc lamp and a 1 cm quartz sample cell. Sample component masses were determined using a MonoPan electronic balance (AB 104, Mettler Toledo, Switzerland), and the medium’s pH was measured with a calibrated Oakton pH 700 m (Vernon Hills, IL 60,061, USA).

### Materials and reagents

Materials were sourced as follows: Plegica® eye drops (1% CYP) from Elezaby Pharmacy; authentic CYP powder (99.97% purity) from Hikma (Egypt); Erythrosine 99.48% w/w precursor from Alpha Chemika (India); organic solvents from Elgomhoria Company (Cairo, Egypt); NaOH, phosphoric acid, HCl, and citric acid from El-Nasr Chemical Company (Cairo, Egypt). For the synthesis, 30 mg of erythrosine precursor was vortex-mixed with 15 mL distilled water and then diluted with water to 100 mL, yielding a 0.03% w/v solution. Torell buffer systems for pH control were formulated from 0.1 M solutions of NaOH, H₃PO₄, citric acid, and HCl, adjusted to the required pH.

### CYP stock preparation

A stock solution of CYP (100 μg/mL) was prepared by dissolving 10.0 mg of the compound in distilled water within a 100.0 mL volumetric flask. Diluted standard solutions spanning concentrations of 0.4 to 15 μg/mL were then generated from this stock using distilled water as the diluent. Aliquots (1 mL) of these standard solutions were employed in the experimental procedures.

### General assay procedure

Experimental samples were prepared in 10 mL volumetric flasks. Each flask received 1 mL of a CYP standard solution (0.4—15 µg/mL), followed by 1.3 mL of 0.03% w/v erythrosine and 1.2 mL of Torell buffer (pH 3.8). The mixture was diluted to the 10 mL mark with distilled water. RRS spectra were recorded at wavelength of 355.5 using synchronous spectrofluorimetric mode. All measured RRS intensities underwent blank correction using a concurrently prepared reagent blank. A calibration curve was generated by plotting the RRS intensity against CYP concentration.

### Assay steps of eye drops

An aliquot of Plegica® 1% Eye Drops corresponding to 20.0 mg CYP was transferred to a 100.0 mL volumetric flask and diluted to volume with distilled water. The solution was sonicated for 10 min. Subsequent dilution with distilled water produced a working solution whose CYP concentration fell within the analytical method’s linear range. The general assay protocol, identical to that applied to calibration standards, was then performed on the working solution. Finally, the percent recovery of CYP was computed employing the pre-determined regression equation from the calibration plot.

## Result and discussion

Xanthene-based fluorophores and their structural analogs have become focal points in analytical chemistry due to their pronounced affinity for biomolecules and their versatile signaling properties. Among these, erythrosine—a polyhalogenated derivative of the fluorone scaffold—has been widely employed as a sensitive indicator for the detection of basic pharmaceutical compounds^24^. When erythrosine encounters an amine-containing drug under suitable conditions, it undergoes an acid–base interaction that yields a novel chromogenic species. Depending on the system’s specifics, this interaction can either amplify Rayleigh scattering signals or attenuate the dye’s native fluorescence emission^25^.

The core of the assay lies in the formation of a 1:1 ion-associate complex. In mildly acidic media, one of erythrosine’s acidic functional groups dissociates, predominantly the phenolic hydroxyl, generating a monovalent anion. Concurrently, the drug’s tertiary amine undergoes protonation, furnishing a positively charged cationic species. Electrostatic attraction between these oppositely charged ions drives complex assembly. Electrostatic forces can manifest either through direct ion–ion contact or via a solvent-mediated shell of counterions, but in this system, the association is reinforced by hydrophobic interactions. Nonpolar regions of both the dye anion and the drug cation congregate to minimize exposure to the aqueous environment, further stabilizing the ion-pair. Once formed, the CYP–erythrosine complex exhibits dramatically enhancement of RRS. The degree of enhancement correlates linearly with drug concentration, enabling precise calibration curves and low limits of detection. This quantitative relationship underpins the assay’s analytical robustness.

**Fig. 1:**
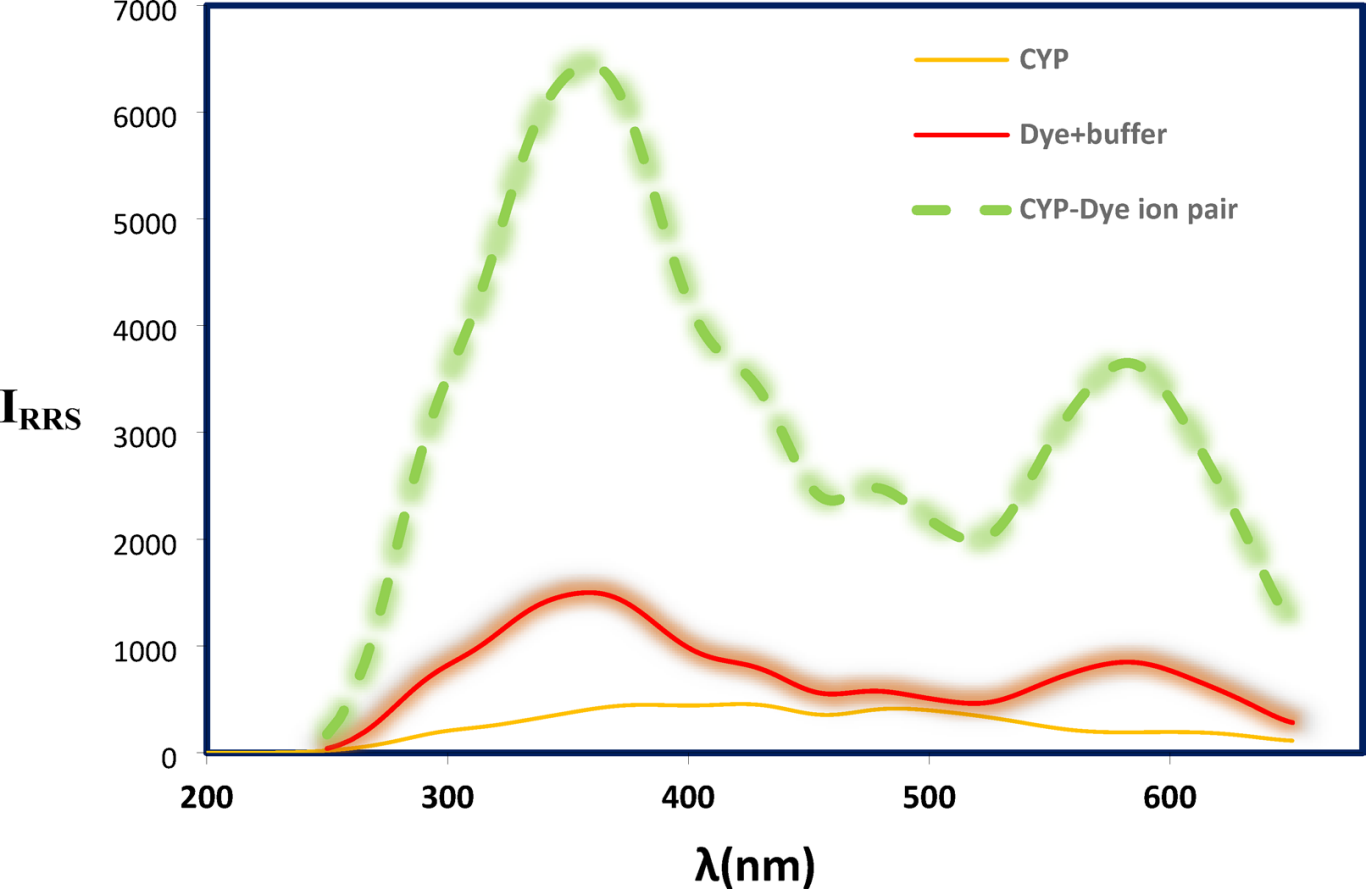
RRS spectra of CYP, erythrosine dye and its reaction product with CYP.

The RRS methodology exploits the enhanced RRS intensity of erythrosine observed at 355.5 nm using synchronous spectrofluorimetry **(**Fig. 1**).** This enhancement arises from increased molecular volume, formation of hydrophobic interfaces, and enhanced molecular rigidity. A principal advantage of this RRS approach is its operational simplicity. There are no protracted extraction steps, no requirement for hazardous organic solvents, and no need for elevated temperatures. The entire procedure can be completed in a single mixing step, aligning well with green chemistry principles.

### Conditions study and tuning

A series of experimental investigations was undertaken to optimize the spectroscopic methodology presented herein, systematically evaluating and refining key reaction parameters influencing the analytical outcomes.

#### pH and buffer volume

**Fig. 2:**
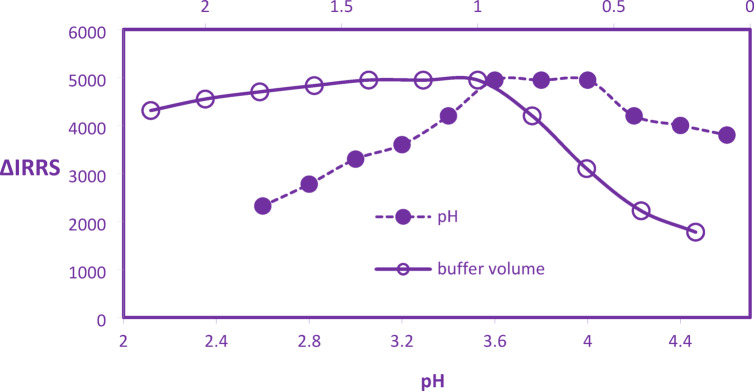
Impact of various pH and buffer volumes in CYP-Dye complex formation.

RRS spectral analysis was employed to investigate the influence of acidic pH on the ion-pair complexation between CYP and erythrosine. The results demonstrated a pronounced pH dependence for complex formation, with maximum RRS signal intensity observed within the pH range of 3.6 to 4.0 **(**Fig. 2**).** This pH optimization significantly enhanced analytical sensitivity, leading to the identification of pH 3.8 as the optimal condition. Furthermore, the effect of TS buffer volume (0.2–2.2 mL) on complex development was assessed via RRS. Signal intensity proved sensitive to buffer quantity, exhibiting maxima between 1.0 and 1.4 mL. Deviations from this range diminished the RRS response; excessive buffer increased ionic strength, impeding electrostatic interaction between cationic CYP and anionic erythrosine and thereby hindering complexation. Conversely, moderate buffer volumes effectively maintained the requisite pH. Consequently, a buffer volume of 1.2 mL was selected for optimal RRS detection **(**Fig. 2**).**

#### Erythrosine volume

**Fig. 3:**
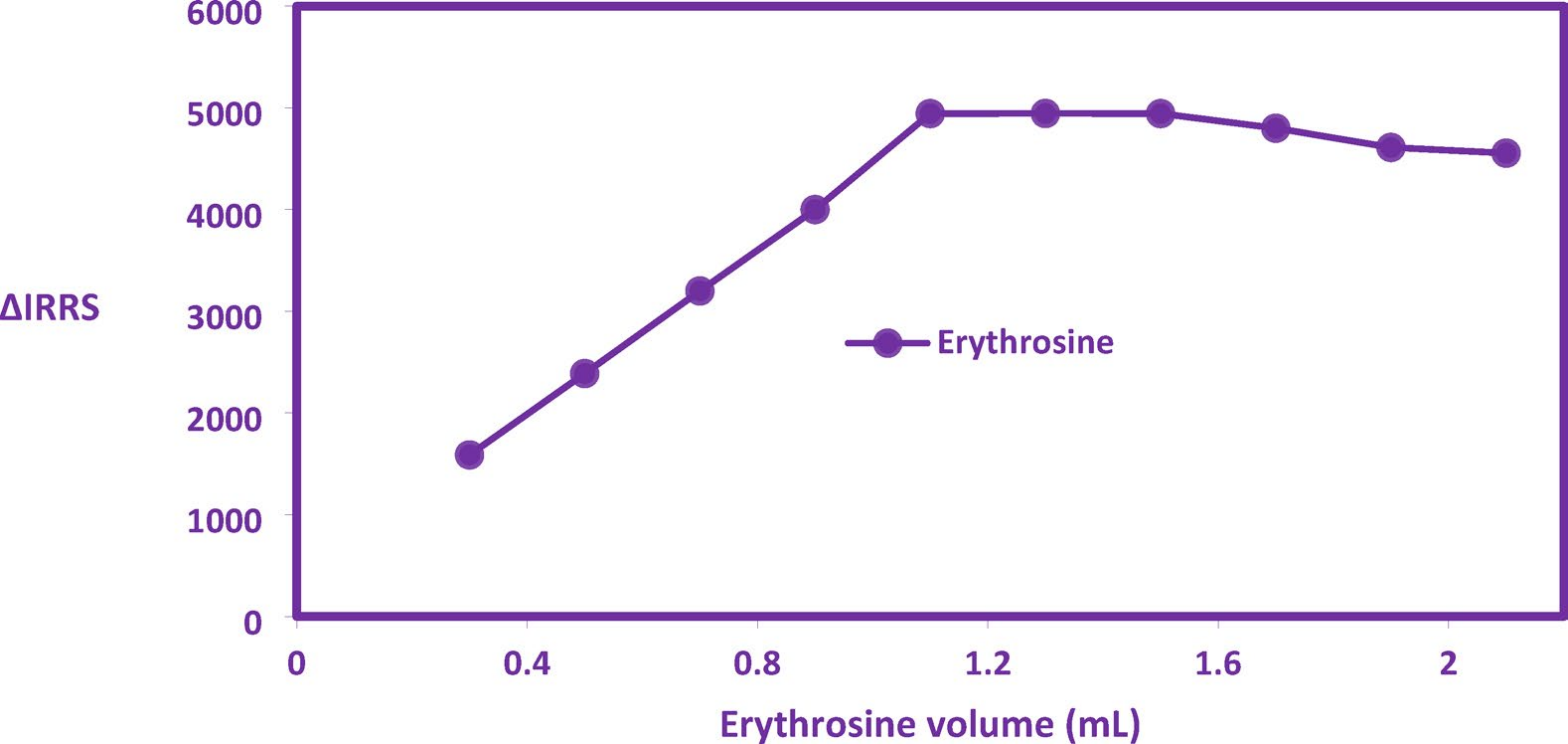
Effect of different volumes of erythrosine dye on the intensity of RRS of the formed complex.

The optimization of erythrosine ligand concentration for RRS detection was investigated. RRS spectral analysis revealed that maximum signal intensity for the CYP-erythrosine ion-associate complex occurred when using 0.03% (w/v) erythrosine solution volumes between 1.1 and 1.5 mL (Fig. 3). Consequently, 1.3 mL was selected as optimal. Suboptimal ligand volumes (< 1.0 mL) provided insufficient dye for complete complexation, diminishing RRS response. Conversely, volumes exceeding 1.5 mL induced concentration-dependent quenching, attributed to erythrosine self-aggregation at higher concentrations, which impeded complex formation and reduced RRS intensity, as evidenced in Fig. 3**.**

#### Dispersing liquid impact

**Fig. 4:**
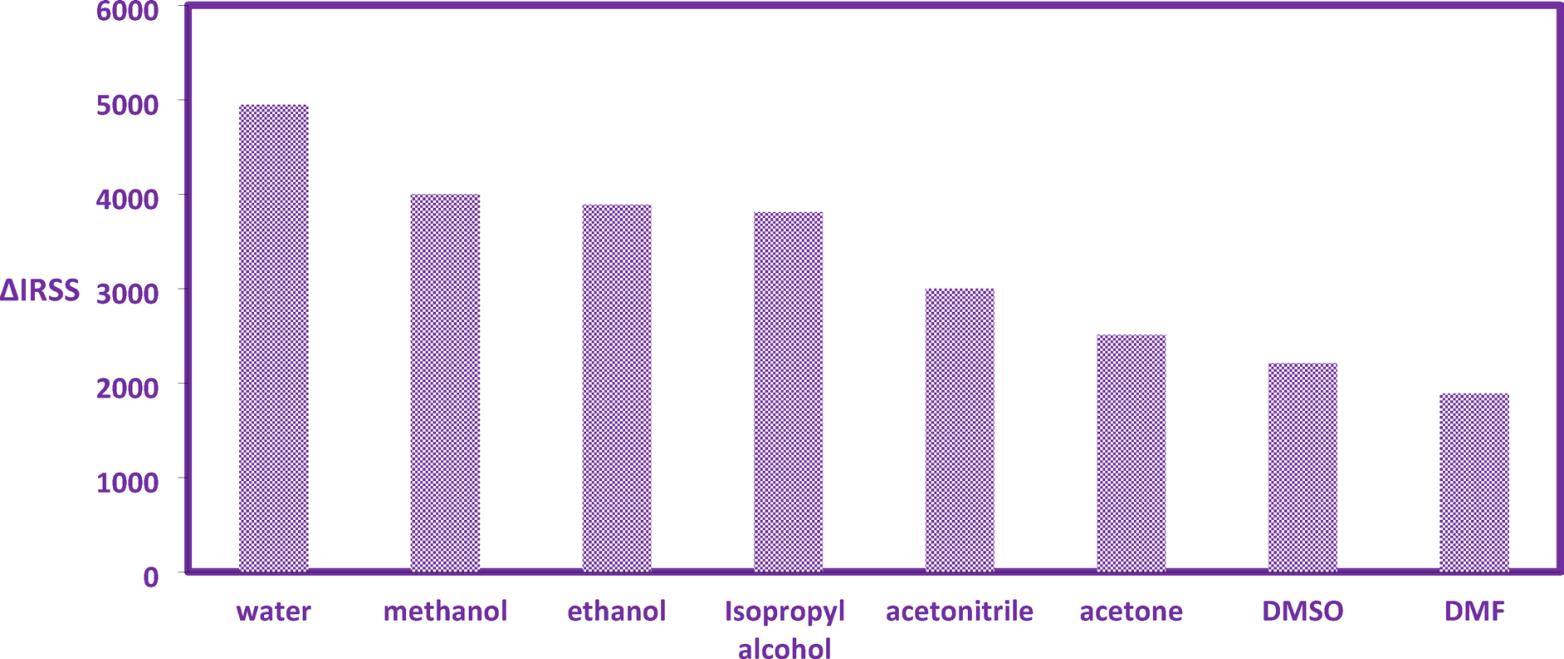
Impact of various dilution solvents on CYP-erythrosine dye complex formation.

The influence of solvent medium on the RRS signal intensity of the CYP-erythrosine complex was evaluated, comparing acetone, distilled water (DW), dimethylformamide (DMF), methanol, ethanol, acetonitrile, isopropyl alcohol, and DMSO **(**Fig. 4**).** RRS analysis revealed that DW induced the most significant signal enhancement while simultaneously offering environmental sustainability. In contrast, organic solvents consistently yielded diminished RRS responses. This attenuation is attributed to solvent-induced disruption of the ion-pair complex formation, potentially degrading electron transfer viability within the complex structure. Consequently, DW was selected as the optimal dispensing solvent for subsequent RRS studies.

### Method validation

Validation of the RRS spectroscopy-based method was conducted according to ICH Q2(R1) requirements^26,27^. Key validation characteristics assessed included linearity across the working range, accuracy (through recovery studies), precision (repeatability and intermediate precision), and robustness against experimental fluctuations. Additionally, the method’s sensitivity was established by calculating the LOD and LOQ.

#### Linearity and range

The developed RRS spectroscopic method was applied to analyze CYP standard solutions. A calibration graph was constructed by plotting the enhancement in RRS intensity against CYP concentration (ng mL^−1^). The method demonstrated linearity over a concentration range of 40 to 1500 ng mL^−1^. Statistical analysis **(**Table 1**)** confirmed a strong linear relationship, evidenced by correlation and determination coefficient values approaching unity.

**Table 1:** Numerical data used to justify and support the development of the system.

Parameters	Values
Linear range (ng/mL)	40–1500
Slope	4.8
%RSD of the slope	51.69%
Variance of the slope	6.34
Intercept	1247.9
SD of intercept (S_a_)	19
Correlation coefficient (r)	0.9998
Determination coefficients (r^2^)	0.9996
Number of determinations	8
Limit of quantitation (ng/mL)	39.5
Limit of detection (ng/mL)	13

#### Accuracy and precision

The accuracy of the RRS method was validated using quality control (QC) samples at 200, 500, 750, and 1250 ng mL⁻^1^ CYP. Quantitative recoveries (≈100%), low standard deviations (SD < 2), and minimal percent error confirmed method accuracy **(**Table 2**).** Precision was evaluated through within-day (intra-day) and between-day (inter-day) analyses at three QC levels. Both %RSD and SD values remained below 2%, demonstrating excellent repeatability and intermediate precision for the RRS-based quantification **(**Table 3**).**

**Table 2:** The accuracy of the designed system across four different concentrations of CYP.

Conc. level (ng/mL)	Recovery* ± SD	% Error
200	99.39 ± 0.45	0.61
500	98.84 ± 0.83	1.16
750	100.39 ± 0.77	0.39
1250	101.07 ± 0.99	1.07

*mean of three CYP determinations and SD is the standard deviation.

**Table 3:** Precision of the developed RRS approach on two levels.

Conc. level (ng/mL)	Intraday precision		Interday precision	
	Recovery* ± SD	RSD	Recovery* ± SD	RSD
300	99.20 ± 1.46	1.47	98.87 ± 1.74	1.75
800	100.29 ± 0.48	0.47	101.96 ± 1.04	1.02
1300	99.13 ± 0.33	0.34	98.05 ± 0.68	0.69

*mean of three CYP determinations and RSD is the relative standard deviation.

#### Robustness

Minor deliberate alterations to operational conditions (pH, buffer volume, erythrosine volume) were employed to test RRS method robustness. Analysis of recovery (%) and SD data revealed consistently high recoveries and low variability. The minimal effect on RRS response demonstrates the method’s stability against minor procedural deviations, as detailed in Table 4**.**

**Table 4:** Assessing the robustness of the current RRS technology for CYP assay.

Parameter	Value	% Recovery* ± SD
pH	3.7	100.11 ± 1.25
	3.9	101.65 ± 1.41
Buffer volume (mL)	1.1	98.01 ± 1.11
	1.3	98.96 ± 1.63
Dye volume (mL)	1.2	99.48 ± 1.16
	1.4	101.43 ± 1.07

*Mean of three CYP replicate measurements, SD = Standard deviation.

### Reaction stoichiometry

**Fig. 5:**
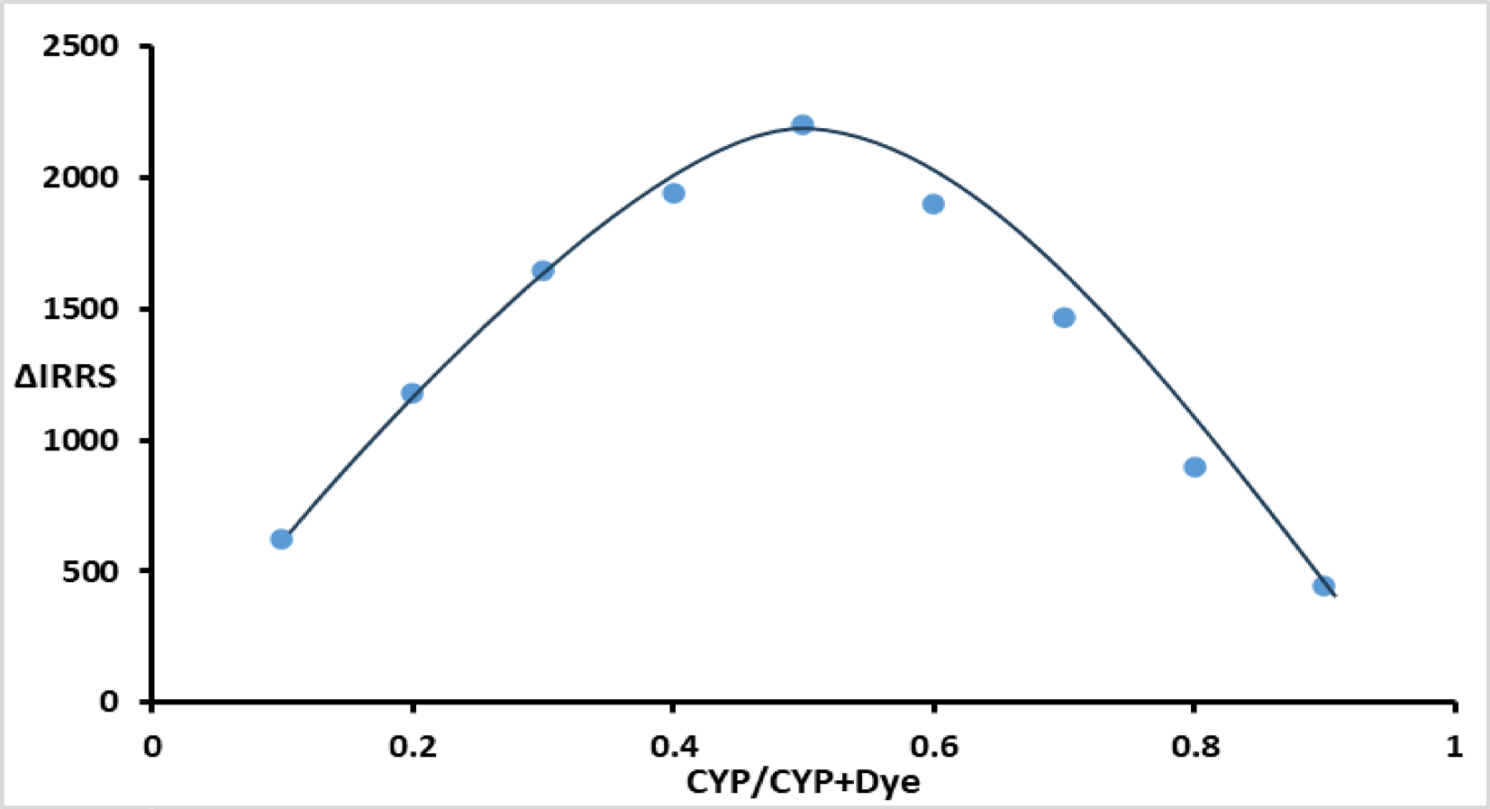
Job’s plot for molar ratio calculation between CYP and dye.

**Fig. 6:**
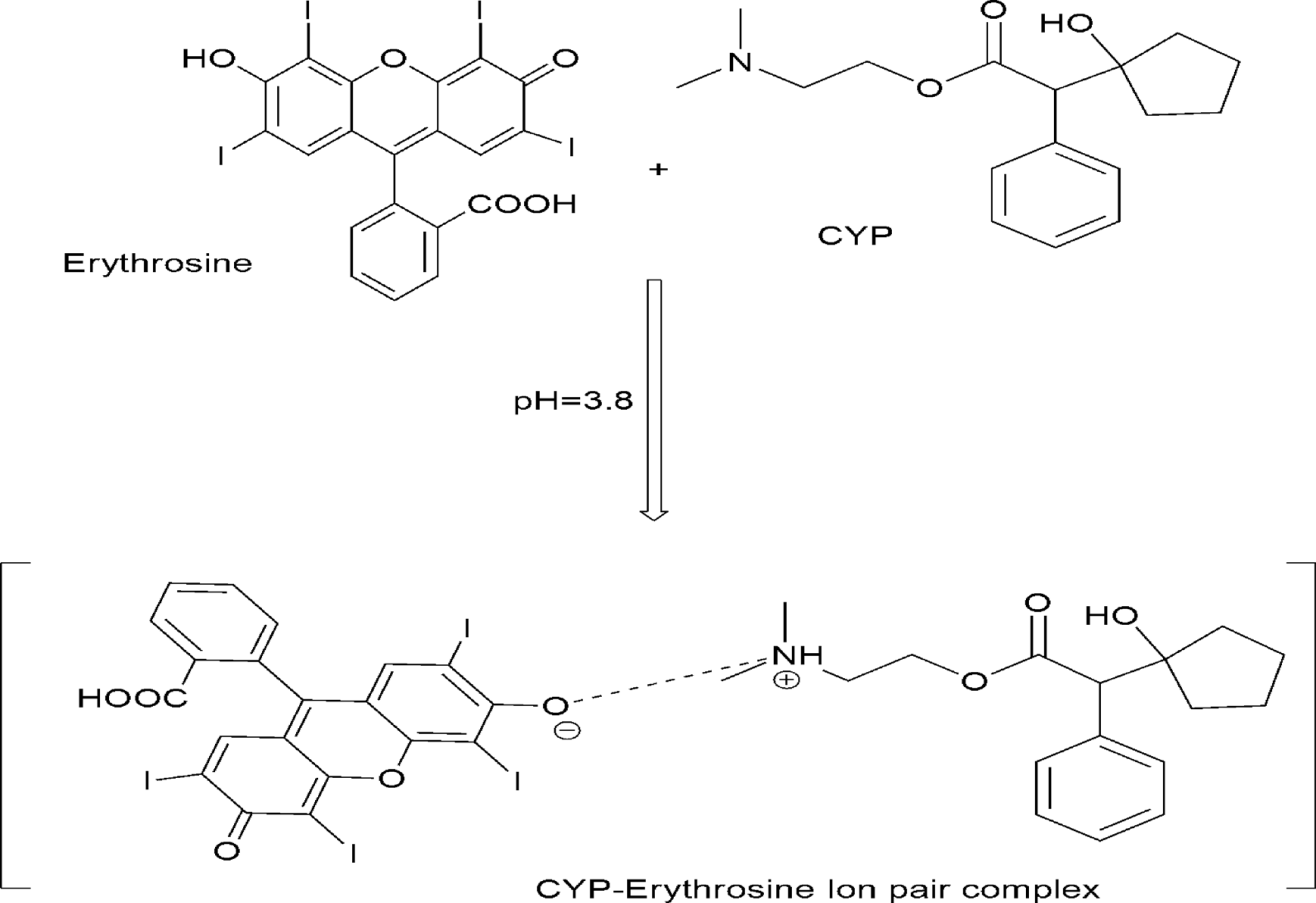
The suggested pathway for the reaction of CYP and erythrosine dye.

The stoichiometry of the erythrosine B-CYP complex was determined via Job’s method of continuous variation using RRS spectroscopy. Equimolar solutions of dye and drug were mixed in complementary volume ratios following the analytical protocol. The enhancement in RRS intensity at 355.5 nm was measured and plotted against the CYP mole fraction. The maximum ΔIRRS occurred at a mole fraction of 0.5 **(**Fig. 5**),** confirming a 1:1 binding stoichiometry for the binary complex as elucidated by Fig. 6**.**

Based on its established acid–base equilibria, where the reported pKa values are approximately pKa₁ ≈ 3.8, pKa₂ ≈ 4.6, and pKa₃ ≈ 9.9, erythrosine B exists in a weakly acidic solution primarily as its monovalent anion (HB^−^); this specific protonation state is a consequence of the strong electron-withdrawing character of the iodine atoms adjacent to the phenolic hydroxyl group, which decreases the electron density on the oxygen atom and consequently favors its dissociation over that of the carboxylic acid group^28^. Simultaneously, in the same protogenic environment, the tertiary amino moiety present in the cyclopentolate molecule is readily protonated to form a cationic species carrying a localized positive charge. The subsequent interaction is driven by the electrostatic attraction between the oppositely charged ions: the anionic erythrosine B species (with its charge predominantly localized on the ionized phenolate oxygen) and the cationic cyclopentolate ammonium ion. This initial ion-coupled association is further reinforced and stabilized by secondary hydrophobic interactions between the non-polar regions of the drug and the xanthene nucleus of the dye, culminating in the formation of a stable, stoichiometric 1:1 binary complex^29,30^.

### Greenness assessment

The principles of green chemistry are increasingly paramount in analytical science, driven by the need to mitigate the environmental footprint of chemical processes^31,32^. As defined by the Environmental Protection Agency, green chemistry involves designing chemical products and procedures that eliminate hazards to human health and the environment, thereby preventing pollution at its source. A primary strategy for achieving this is the adoption of benign, renewable reaction media—such as water, ionic liquids, or supercritical fluids—to replace conventional toxic solvents. This commitment to sustainability underpins the development of the green instrumental methodology presented herein for the rapid determination of CYP.

**Fig. 7:**
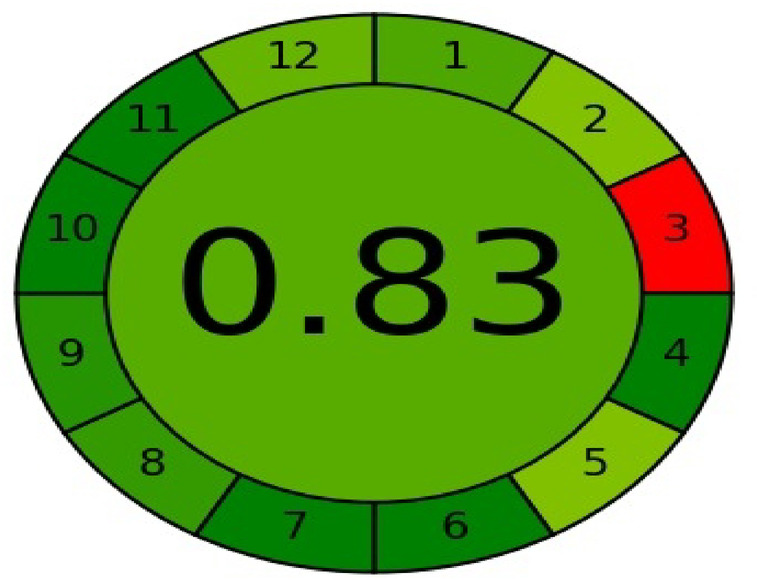
The greenness evaluation of the planned method using AGREE ecological metrics.

The growing emphasis on ecological responsibility has spurred the creation of dedicated assessment tools to evaluate the environmental impact of analytical methods. This evaluation is critical, as standard techniques can generate significant waste; for instance, a single HPLC system typically produces roughly 0.5 L of organic solvent waste daily^33,34^. Consequently, a formal greenness assessment has become an essential component of method validation. The AGREE metric exemplifies this approach, employing a clock-shaped pictogram where each of its 12 segments corresponds to one of the 12 principles of green analytical chemistry (GAC). The overall greenness score, displayed at the center, ranges from 0 to 1, with scores approaching 1 indicating a superior environmental profile. As depicted in Fig. 7, the proposed method achieved a high score, confirming its minimal ecological impact.

## Application

Commercial *Plegica*® eye drops were analyzed using the optimized RRS method to assess CYP content. Statistical equivalence to the reference method^7^ was validated via *t*-tests (accuracy) and *F*-tests (precision) of recovery data. Results **(**Table 5**)** demonstrated *t*- and *F*-values below theoretical thresholds, proving the RRS approach is statistically indistinguishable from the conventional method in both accuracy and precision. The Resonance Rayleigh Scattering (RRS) spectroscopy method developed in this work demonstrates significant analytical advantages for pharmaceutical quantification. Its exceptional sensitivity enables precise detection of target analytes at relevant concentrations across a broad dynamic range. The technique’s environmental sustainability is evidenced by the optimal use of aqueous media, eliminating hazardous organic solvents while maintaining signal integrity. Rigorous validation confirms the outstanding robustness of the method against deliberate operational variations, ensuring methodological reliability under routine laboratory conditions. Furthermore, statistical equivalence to established reference methods was demonstrated through comprehensive hypothesis testing, validating its accuracy and precision for commercial pharmaceutical formulations. The approach’s mechanistic specificity is highlighted by definitive stoichiometric characterization of the complex formation. Collectively, these attributes establish RRS spectroscopy as a green, robust, and reliable alternative to conventional analytical techniques for quality control applications.

**Table 5:** Dosage form analysis of CYP and comparison with the reported method.

Dosage form	Current method	Reported method		
	Recovery^a^ ± SD	Recovery^a^ ± SD	t‐ test value^b^	F‐value^b^
Plegica 1% Eye drop	98.81 ± 1.72	98.550 ± 1.234	0.28	1.94

^a^The value is the average of five determinations for both the proposed and reported methods.^b^Tabulated values at 95% confidence limit are t = 2.306, F = 6.338.

The current methodology exhibit remarkable sensitivity compared to reported method^7^.

- Current method linear range, LOD are 40–1500 and 13 ng / mL
- Reported method linear range, LOD are 20–500 and 6 µg / mL

## Conclusion

The developed Resonance Rayleigh Scattering (RRS) spectroscopic method for CYP analysis exemplifies an environmentally sustainable, robust, and rapid analytical approach. This technique exploits the RRS signal enhancement arising from ion-associate complex formation between CYP and erythrosine, enabling straightforward quantitative determination in ophthalmic formulations without costly instrumentation. Comprehensive optimization of experimental parameters governing complexation and RRS enhancement was undertaken. A critical advantage of the erythrosine-based methodology is the immediate complex assembly, significantly reducing analysis time and simplifying procedural workflow. Furthermore, erythrosine exhibits a favorable safety profile relative to conventional chromogenic reagents, aligning with green chemistry principles increasingly prioritized in modern analytical science. The assay eliminates labor-intensive, environmentally burdensome steps such as solvent extraction or thermal activation, thereby enhancing operational safety and reducing ecological impact. Collectively, these attributes—rapidity, operational simplicity, reduced environmental footprint, and inherent safety—establish the erythrosine RRS approach as a viable green alternative for sensitive CYP quantification.

## Data Availability

The data will be available upon reasonable request from the corresponding author.
